# A Rare Case of Isolated Intramedullary Spinal Cord Cysticercosis

**DOI:** 10.7759/cureus.14864

**Published:** 2021-05-06

**Authors:** Abhishek Vadher, Maharshi R Raval, Suchi D Shah, Kishan G Patel, Kamal Sharma

**Affiliations:** 1 Internal Medicine, B.J. Medical College, Ahmedabad, IND; 2 Internal Medicine, Heart and Vascular Institute, Dearborn, USA; 3 Internal Medicine, AMC Medical Education Trust Medical College, Ahmedabad, IND; 4 Department of Cardiology, U N Mehta Institute of Cardiology and Research Centre, Ahmedabad, IND

**Keywords:** neurocysticercosis, spinal cysticercosis, intramedullary, tinea solium, parasitic infection

## Abstract

Neurocysticercosis is a parasitic disease often involving the central nervous system by *Taenia solium* and is commonly seen in developing countries. The majority of these cases have either isolated brain involvement or combined involvement of the brain and spinal cord. Isolated involvement of the spinal cord is very rare. We report the case of a 20-year-old Indian man who was hospitalized for progressive weakness in all extremities. Magnetic resonance imaging showed a well-defined, round, thick-walled, peripherally enhancing lesion in the intramedullary region, a provisional diagnosis of isolated cysticercosis of the intramedullary region of the spinal cord was made. The patient improved upon needle aspiration of the cystic lesion after surgery, which on post-surgical histological examination confirmed the diagnosis by showing the presence of cysticerci.

## Introduction

Cysticercosis is the infection caused by the larval stage of pork tapeworm, *Taenia solium*. The definitive host are humans and the intermediate host can be animals such as pigs. When an animal consumes eggs excreted in human feces, the larva gets encysted into the tissues. When humans consume these larvae by eating undercooked animals, they may get an intestinal infection [1]. After ingestion, embryos are released in the intestinal lumen and larvae invade the intestinal wall which then disseminates hematogenously to encyst the human brain, skeletal muscle, subcutaneous tissue, and eyes, with the brain being the most common site (60-90%) and eyes being the least common site (1-3%) [2]. Intramedullary spinal cord cysticercosis is a rare entity and may encounter diagnostic difficulty owing to the fact that its imaging findings are similar to other differential diagnoses.

## Case presentation

**Figure 1:**
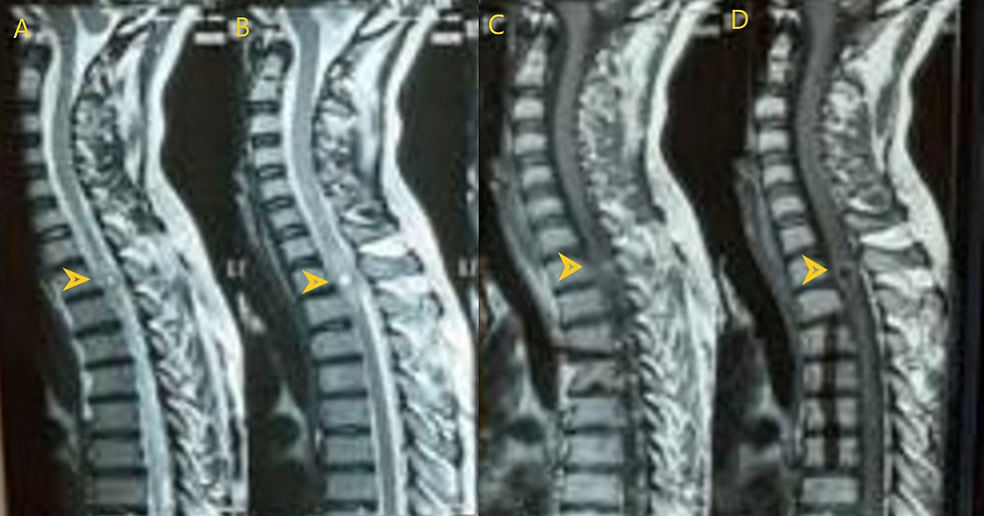
Cervico-dorsal spinal MRI. (A) Short TI-inversion recovery image. (B) T2-weighted image. (C) Post-contrast T1-weighted image. (D) Plain T1-weighted image. A and B show a well-defined, thick-walled, hyperintense lesion. C and D show a hypointense lesion on T1-weighted image with post-contrast ring enhancement. MRI: magnetic resonance imaging

A 20-year-old male patient presented with complaints of progressive, ascending weakness in both upper and lower limbs over a period of two weeks. The weakness started in the left lower limb and then progressed to the right lower limb followed by gradual and simultaneous weakness of the left upper limb and the right upper limb. He also complained of bowel and bladder incontinence for the last five days. On neurological examination, muscle power was 3/5 in all four limbs. Muscle tone was increased and there were brisk deep tendon reflexes in all limbs. Babinski’s sign was positive bilaterally. The patient was not able to walk even with support. He had reduced sensations to pain and temperature below the level of T1. No significant anomaly was found in vibration, proprioception, and tactile sensation in all four limbs. Vitals, general examination, and systemic examination including respiratory function were unremarkable. In view of the brisk deep tendon reflexes and positive Babinski’s sign bilaterally, a differential diagnosis of ascending paralysis (Guillain-Barré syndrome) was clinically ruled out. Computed tomography scan of the brain was performed and was unremarkable, ruling out the diagnosis of cerebrovascular accident. A lesion in the cervical spine was suspected clinically. Magnetic resonance imaging (MRI) of the cervico-dorsal spine with whole spine screening was done which showed a single well-defined, round, thick-walled, peripherally enhancing lesion in the intramedullary region of the spinal cord at the level of T1 vertebral body with associated swelling of the cord in the cervico-dorsal region from C7 to T2 along with diffuse T2-weighted image (T2WI) hyperintensity within the cord, mainly in the posterior aspect of the cord showing possible cord edema, suggestive of possibly granulomatous lesion with cord edema (Figure 1). As cysticercosis is common in India, a diagnosis of cysticercosis of the spinal cord was considered to be the most likely etiology.

**Figure 2:**
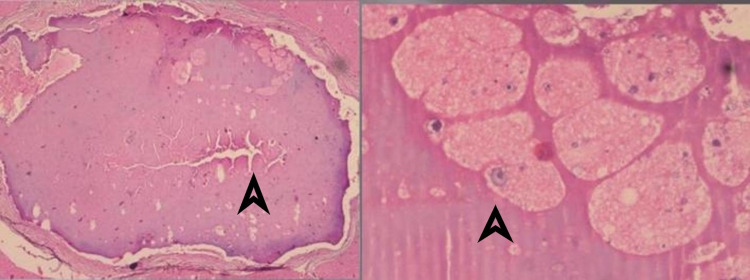
Biopsy of the resected section. Right: cystic structures of cysticercosis. Left: convoluted spiral canal of the parasite.

The patient underwent laminectomy from C7 to T2 which showed a swollen spinal cord. On performing midline myelotomy, white cystic lesions were seen which on aspiration showed clear fluid. The resected section was sent for histological examination which showed cystic structures of cysticercosis surrounded by inflammatory cells. The parasites’ main structural features include a prominent investing tegument or cuticle, aggregated subcuticular cells, smooth muscle fibers, and four suckers, which were also seen on histological examination (Figure 2). Postoperatively, the patient was given oral steroids and albendazole for a period of four weeks, following which the patient was discharged. Follow-up at six months showed improvement in motor function. The muscle power improved to 4+/5 in all four limbs. The patient was able to walk with minimal support. He regained bowel and bladder functions but there was no improvement in pain and temperature sensation loss over C7-T2 dermatomes.

## Discussion

Neurocysticercosis is the most common parasitic infection of the central nervous system (CNS). Isolated spinal cord cysticercosis is rare and represents only 1-3.2% of all cases of neurocysticercosis [3,4]. Spinal cysticercosis is classified as extraspinal (vertebral) or intraspinal, including epidural, subdural, arachnoid, or intramedullary. Isolated intramedullary infection is very rare and fewer than 100 cases have been reported worldwide [5-9]. Pain, paraparesis, quadriparesis, spasticity, bowel, bladder incontinence, and sexual dysfunction are common manifestations of spinal cord cysticercosis [4]. The factors that explain the development of the clinical symptoms are: (1) cyst causing mechanical compression, (2) degeneration of the larva remnants and the subsequent inflammation, and (3) gliosis [10].

There are two hypotheses to explain the mode of entry of the parasite into the spinal cord. One hypothesis suggests that the parasite spreads from blood circulation. The thoracic spinal cord has a relatively higher blood supply when compared to other regions of the spinal cord, and hence, most cases of spinal cord cysticercosis are seen involving the thoracic spinal cord. The second hypothesis suggests that the spread of the parasite to the spinal cord is through the ventriculo-ependymal pathway [4]. The differential diagnosis of intramedullary cysticercosis can be an arachnoid cyst, ependymal cyst, sarcoidosis, neurenteric cyst, abscess, or ependymoma [11-13].

CNS cysticercosis affects men and women equally [14]. The peak incidence is seen in the third and fourth decades of life [14]. Clinical manifestations of spinal cysticercosis depend on the number of lesions and the location of the lesions. The patient’s individual immune response also plays a pivotal role in the clinical manifestations of the disease. Cerebrospinal fluid examination may provide reliable evidence of inflammation, and immunoblot tests may be useful in diagnosing cysticercosis [15]. MRI is a very useful tool in diagnosing as well as describing the location and the stage of the lesion. MRI findings include the presence of a cyst with an eccentric mural nodule representing the scolex showing a hypointense rim with a hyperintense core on T2W1 and a hypointense or isointense lesion on T1W1. These findings are described in detail by Mathuriya et al. [16]. The findings are only suggestive and not diagnostic as it may be seen in other conditions such as a neurenteric cyst and abscess. MRI whole spine screening with MRI brain is recommended in all patients of spinal cysticercosis to detect any additional lesions.

As cysticercosis has a pleomorphic nature, there are multiple approaches to management. The two mainstays of the management are medical and surgical interventions. The location of the parasite and the activity of the disease are the main factors that guide the treatment of spinal cysticercosis. Surgical intervention is recommended when there is a doubtful diagnosis or the presentation of the symptoms is acute. Surgical intervention not only provides decompression but also provides tissue for histopathological examination which aids in the diagnosis [6]. The results of surgical interventions vary to a great extent and depend solely on a case-by-case basis. Studies have shown that 60-87.5% of patients have satisfactory outcomes after surgery with significant improvement when combined with medical treatment with albendazole and steroids. A study by Ahmad et al. reported a 60% improvement in the cases [4]. The case series reported by Mohanty et al. reported recovery in 87.5% of cases [17]. Generally, albendazole is given postoperatively at a dose of 15 mg/kg/day for four to six weeks as cysticercosis is a generalized disease with the manifestation of the spinal cord being only a focal manifestation of the systemic disease. Albendazole is given along with steroids to reduce edema which might develop as the parasite dies. There is also a minor role of albendazole preoperatively as it facilitates the consolidation of the lesion [18].

## Conclusions

We report a rare case of isolated intramedullary spinal cysticercosis. Imaging studies like MRI can aid in diagnosis, but given the rare nature of the condition and nonspecific radiological findings, it may be missed. Histopathological examination of the specimen resected after surgery is often confirmatory. Early diagnosis and treatment can improve the outcome.
